# Pedicle screw placement in spinal neurosurgery using a 3D-printed drill guide template: a systematic review and meta-analysis

**DOI:** 10.1186/s13018-019-1510-5

**Published:** 2020-01-03

**Authors:** Chengqiang Yu, Yufu Ou, Chengxin Xie, Yu Zhang, Jianxun Wei, Xiaoping Mu

**Affiliations:** Department of Orthopaedics, The People’s Hospital of Guangxi Zhuang Autonomous Region, Nanning, Guangxi 530021 China

**Keywords:** Drill guide template, Pedicle screw, Free-hand technique, Pedicle screw fixation

## Abstract

**Background:**

Many surgeons believe that the use of a 3D-printed drill guide template shortens operative time and reduces intraoperative blood loss compared with those of the free-hand technique. In this study, we investigated the effects of a drill guide template on the accuracy of pedicle screw placement (the screw placed completely in the pedicle), operative time, and intraoperative blood loss.

**Materials/Methods:**

We systematically searched the major databases, such as Medline via PubMed, EMBASE, Ovid, Cochrane Library, and Google Scholar, regarding the accuracy of pedicle screw placement, operative time, and intraoperative blood loss. The *χ*^2^ test and *I*^2^ statistic were used to examine heterogeneity. Odds ratios (ORs) with 95% confidence intervals (CIs) were used to calculate the accuracy rate of pedicle screw placement, and weighted mean differences (WMDs) with 95% CIs were utilized to express operative time and intraoperative blood loss.

**Results:**

This meta-analysis included 13 studies (seven randomized controlled trials and six prospective cohort studies) involving 446 patients and 3375 screws. The risk of research bias was considered moderate. Operative time (WMD = − 20.75, 95% CI − 33.20 ~ − 8.29, *P* = 0.001) and intraoperative blood loss (WMD = − 106.16, 95% CI − 185.35 ~ − 26.97, *P* = 0.009) in the thoracolumbar vertebrae, evaluated by a subgroup analysis, were significantly different between groups. The 3D-printed drill guide template has advantages over the free-hand technique and improves the accuracy of pedicle screw placement (OR = 2.88; 95% CI, 2.39~3.47; *P* = 0.000).

**Conclusion:**

The 3D-printed drill guide template can improve the accuracy rate of pedicle screw placement, shorten operative time, and reduce intraoperative blood loss.

## Introduction

The malpositioning of pedicle screws (pedicle screw placement inaccuracy) is one of the most difficult problems encountered in pedicle screw fixation and often leads to a series of complications after surgery, such as nerve root injury, spinal cord injury, and surrounding soft tissue or bone destruction [1, 2]. The incidence of pedicle screw misplacement can be as high as 15~30% in severe spinal deformity surgeries [3]. It is well known that screw misplacement has unpredictable clinical consequences for the patient. Reoperation or removal of the screw not only increases the patient’s financial burden and the pressure on the doctor but also leads to unnecessary waste of medical resources [4]. Therefore, it is very important to enhance the accuracy rate of pedicle screw placement using auxiliary equipment. To improve the accuracy rate of screw insertion, many new technologies have been developed, such as CT navigation and robotics. In addition, it has also been found that the complete insertion of screws in the pedicle is closely related to the surgeon’s proficiency in surgery and postoperative X-ray assessment [5]. The current computer-assisted imaging navigation system used for guiding pedicle screw placement has been used since the end of the twentieth century. In 1999, Kamimura et al. [6] used computer-assisted navigation systems to achieve satisfactory results in both laboratory and clinical settings. Therefore, to enhance clinical outcomes, we should improve the surgical proficiency of spine surgeons and develop more advanced equipment.

Recently, according to the reports of relevant clinical experiments, the drill guide template has been successfully applied in the placement of pedicle screws in spine surgeries [7–9]. Some studies have shown that patient-specific drilling guidance templates can reduce the incidence of pedicle screw misplacement and decrease the operative time and intraoperative blood loss; however, most of these studies involved animal experiments and cadaver specimen experiments [10–12]. Fan et al. [13] compared the screw perforation rates of the two methods, but cadaver experiments were included in their studies, and outcome indicators such as operative time and intraoperative blood loss were not systematically evaluated. In addition, Chen et al. [14] suggested that the drill guide template does not improve the accuracy rate of screw placement compared with that of the traditional method.

In view of the controversies and the inadequacies of current clinical studies, we conducted a meta-analysis of related studies to determine whether the drill guide template can increase the accuracy rate of screw placement as well as whether it decreases the operative time and intraoperative blood loss compared with those of the free-hand technique.

## Methods

### Search

In accordance with the research purpose, two authors (C.Q.Y. and X.P.M.) searched relevant electronic databases, such as Medline via PubMed, EMBASE, Ovid, Cochrane Library, and Google Scholar. The time range of the articles included in the search was from the database establishment date to August 2019. The main keywords were as follows: drill guide template, patient-specific drill template, pedicle screw, free-hand technique, and pedicle screw fixation. The language of the articles was not limited. In addition, we manually searched the relevant literature to determine whether these documents met the inclusion criteria of this study. Finally, we carefully read the full text of the articles that met the inclusion criteria and extracted relevant data for a comprehensive evaluation.

### Inclusion criteria and exclusion criteria

The inclusion criteria of the literature included the following aspects:

1. Studies that included patients with a spinal disease requiring pedicle screw fixation, such as atlantoaxial instability, scoliosis, or severe spinal stenosis;
2. Only clinical studies that included both a template group and a freehand group;
3. Studies that included an observation group who underwent surgery with a screw navigation template containing a drill hole;
4. Studies that included a control group who underwent surgery with a traditional hand-placed screw;
5. Only clinical studies, including clinical randomized controlled trials and retrospective case-control studies.
  When we encountered the following conditions, we excluded the corresponding article.
6. Summaries, letters, case reports, and summaries of meetings;
7. Studies with incomplete or unavailable data;
8. Duplicate publications, studies with animal experiments, and cadaveric experimental studies;
9. Gray literature and articles with poor quality evaluation or low credibility.

### Data extraction and quality assessment

The data were independently extracted by two researchers (C.Q.Y. and Y.F.O) from eligible articles. The data included the following: name of the first author, year of publication, location of the study, year of the study, type of intervention, demographic characteristics (number, sex, and age), number of total screws, number of qualified screws, number of misplaced screws, operative time, and intraoperative blood loss. Since the evaluation criteria for the screw placement level were different among studies, we divided the inserted screws into qualified (screw completely within pedicle) and unqualified categories according to the postoperative CT scan results. The extraction of continuous data, such as operative time and intraoperative blood loss, mainly included the mean and standard deviation (mean, SD).

We used the risk of bias assessment tool provided by the Cochrane Handbook for Systematic Reviews of Interventions [15] to assess the quality of randomized clinical controlled trials. The quality assessment of the RCTs was carried out using the software Review Manager Version 5.1 (Cochrane Collaboration, Software Update, Oxford, UK). The nine-star Newcastle-Ottawa Scale (NOS) [16] was used to evaluate the quality of clinical observational studies. The Newcastle–Ottawa Scale is a validated tool for the quality assessment of nonrandomized studies, including cohort and case-control studies, and contains the following categories: selection of cohorts (four items), comparability of cohorts (one item), and assessments of outcomes (three items). A study can be awarded a maximum of one star for each item within the selection of cohort and assessment of outcome categories and two stars for the items in the comparability of cohort category. The scores of the items identifying study quality varied from zero to nine. Any disagreement was settled by a group discussion with a third investigator.

### Statistical analysis

For the included studies that reported raw dichotomous data, the odds ratios (ORs) with 95% confidence intervals (CIs) were calculated, and for the extracted continuous data, the weighted mean differences (WMDs) with 95% confidence intervals (CIs) were calculated. The heterogeneity between the studies was calculated by the *χ*^2^ test and *I*^2^ statistic. When *P* > 0.1 and *I*^2^ < 50%, there was no heterogeneity, and we used a fixed effects model; otherwise, we used a random effects model to pool the data. In addition, we used a subgroup analysis to assess the robustness of the meta-analysis results. When the eligible studies were adequate (*n* ≥ 10), funnel plots of Begg’s test were examined for the existence of publication bias. All statistical analyses were performed using Stata (version 12.0). All *P* values were two-sided, and *P* < 0.05 was considered statistically significant.

## Results

### Literature search results

**Fig. 1:**
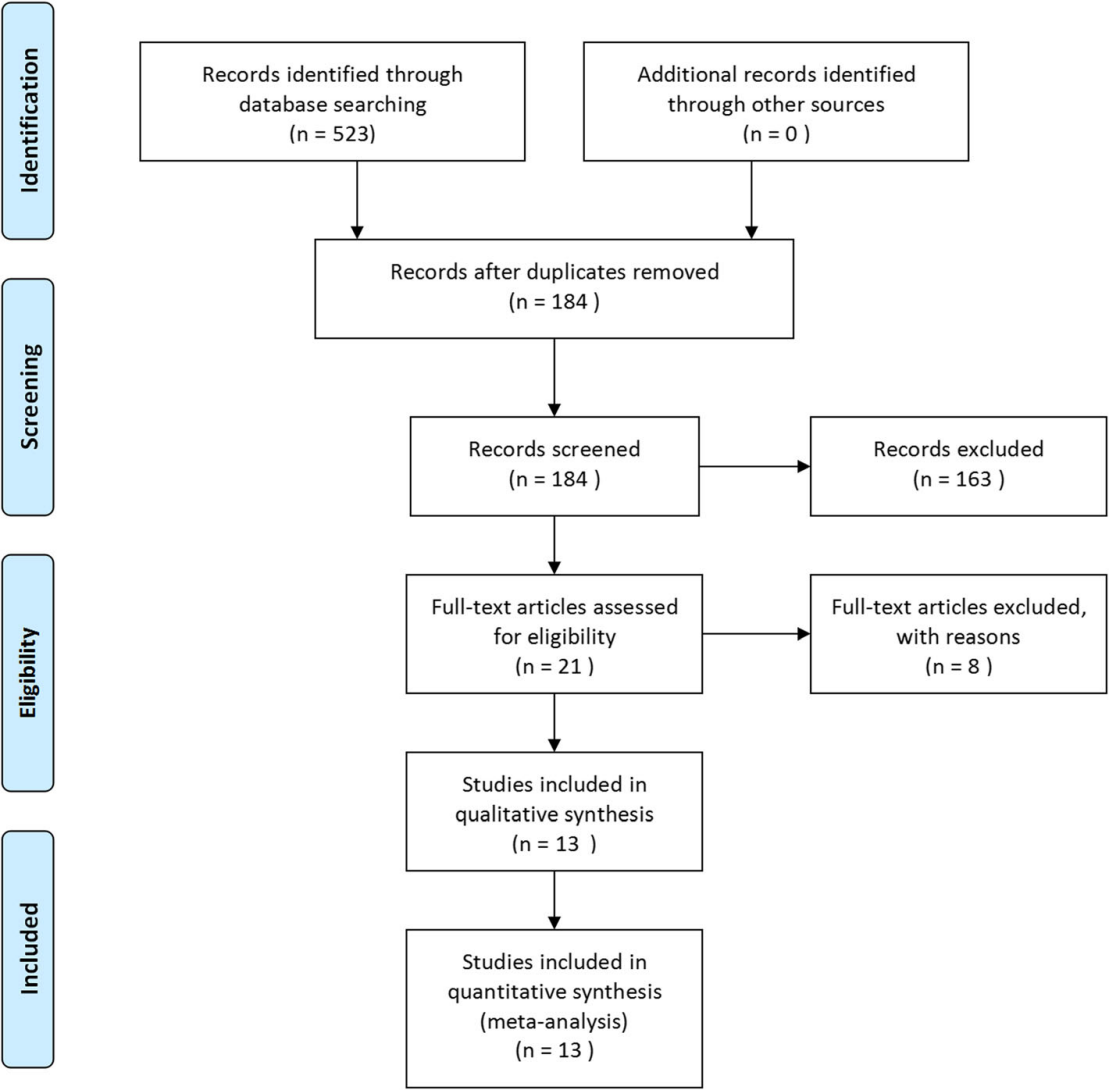
Flow chart

The search strategy initially produced 523 articles, and 184 articles remained after 339 duplicate documents were excluded. Another 163 irrelevant articles were then excluded after the study titles and abstracts were screened. Then, 4 animal studies and 4 cadaver specimen studies were excluded from the 21 studies that met the inclusion criteria. Finally, 13 eligible trials [14, 17–28] were included in this meta-analysis. The flow diagram for the literature selection is shown in Fig. 1.

### Characteristics and quality assessment of the included studies

Table 1 shows the basic characteristics that were independently extracted by two authors (the first and second authors) from the 13 studies. All studies included at least one observational outcome, and the research was recently published. Five studies were published by foreigners, and the other eight studies were written by Chinese researchers. These studies involved a total of 466 patients. A total of 1700 pedicle screws were placed using the drill navigation template, and 1675 pedicle screws were placed using the free-hand technique. Seven studies reported operative time, and five studies reported intraoperative blood loss.

**Table 1:** The characteristics of the extracted research data

Author	Publication year	Study location	Study year	Group	*N*	Age (year), mean (SD)	Male, (male/female)	Number of total screws	Number of qualified screws	Number of misplace screws	Operative time (min), mean (SD)	Blood loss (ml), mean (SD)	Other outcomes
Cecchinato R	2019	Italy	2015.1–2016.10	A B	14 15	34 (15.3) 26 (17.2)	2/12 1/14	297 243	224 160	73 83	UC	UC	④⑤
Chen H	2015	China	2014.8–2015.3	A B	20 23	52.3 (8.75) 55.4 (8.75)	9/11 12/11	118 122	108 99	10 23	UC	UC	④
Garg B	2018	India	UC	A B	10 10	15.5 (3.8) 16.6 (4.9)	3/7 4/6	137 126	125 104	12 22	235.3 (55.7) 298.5 (59.3)	630 (227.6) 840 (533.3)	④⑤
Guo F	2017	China	UC	A B	13*	UC	UC	37 37	32 23	5 14	UC	UC	④⑤
Jiang L	2017	China	2012.6–2014.12	A B	25 29	43.5 (10) 46.9 (7.25)	16/9 18/11	100 116	96 103	4 13	171.84 (22.46) 182.76 (28.4)	309.20 (33.41) 322.07 (26.51)	①③⑤⑧
Liu K	2016	China	2011.10–2015.3	A B	10^*^	UC	UC	48 104	45 82	3 22	UC	UC	⑥
Merc M	2013	Slovenia	2011–2012	A B	9 10	59 (5) 62 (12)	4/5 5/5	54 54	50 45	4 9	143 (113) 176 (90)	UC	**–**
Merc M (2)	2013	Slovenia	UC	A B	7 8	UC	UC	30 36	26 21	4 15	UC	UC	**–**
Merc M	2017	Slovenia	2011–2013	A B	11 13	60.6 (6.7) 62.8 (11.7)	5/6 6/7	72 72	66 43	6 29	UC	UC	①②③
Pan Y	2018	China	2014.1–2017.6	A B	20 17	16.3 (1.8) 16.5 (1.4)	7/13 6/11	396 312	354 234	42 78	283 (22.7) 285 (25.8)	UC	⑥
Pu X	2018	China	2013.6–2016.6	A B	25 24	UC	11/14 14/10	100 96	98 72	2 24	106.1 (10.9) 138.2 (21.7)	185.0 (59.5) 311.0 (87.4)	①③⑧
Wu C	2015	China	2011.2–2013.11	A B	18 24	37.2 (25–57) 38.7 (27–56)	UC	144 192	131 135	13 57	133.3 (15.9) 162.2 (21.7)	735.1 (127.7) 843.2 (147.8)	②④
Zhang Y	2015	China	2013.12–2014.12	A B	10 10	11.9 (4.2) 12.6 (3.8)	3/7 2/8	167 165	138 98	29 67	163.5 (53.7) 164.0 (48.7)	455 (447) 415 (389)	①⑦

*: the total number of participants in both group A and B, *A* template group, *B* free-hand group, *TN* total number, *N* number of patients, *UC* unclear, *SD* standard deviation, ① VAS, ② ODI, ③ follow-up in months, ④ mean time per screw, ⑤ number of fluoroscopy, ⑥ postoperative Cobb angle, ⑦ changes of creatinine level, ⑧ JOA

**Fig. 2:**
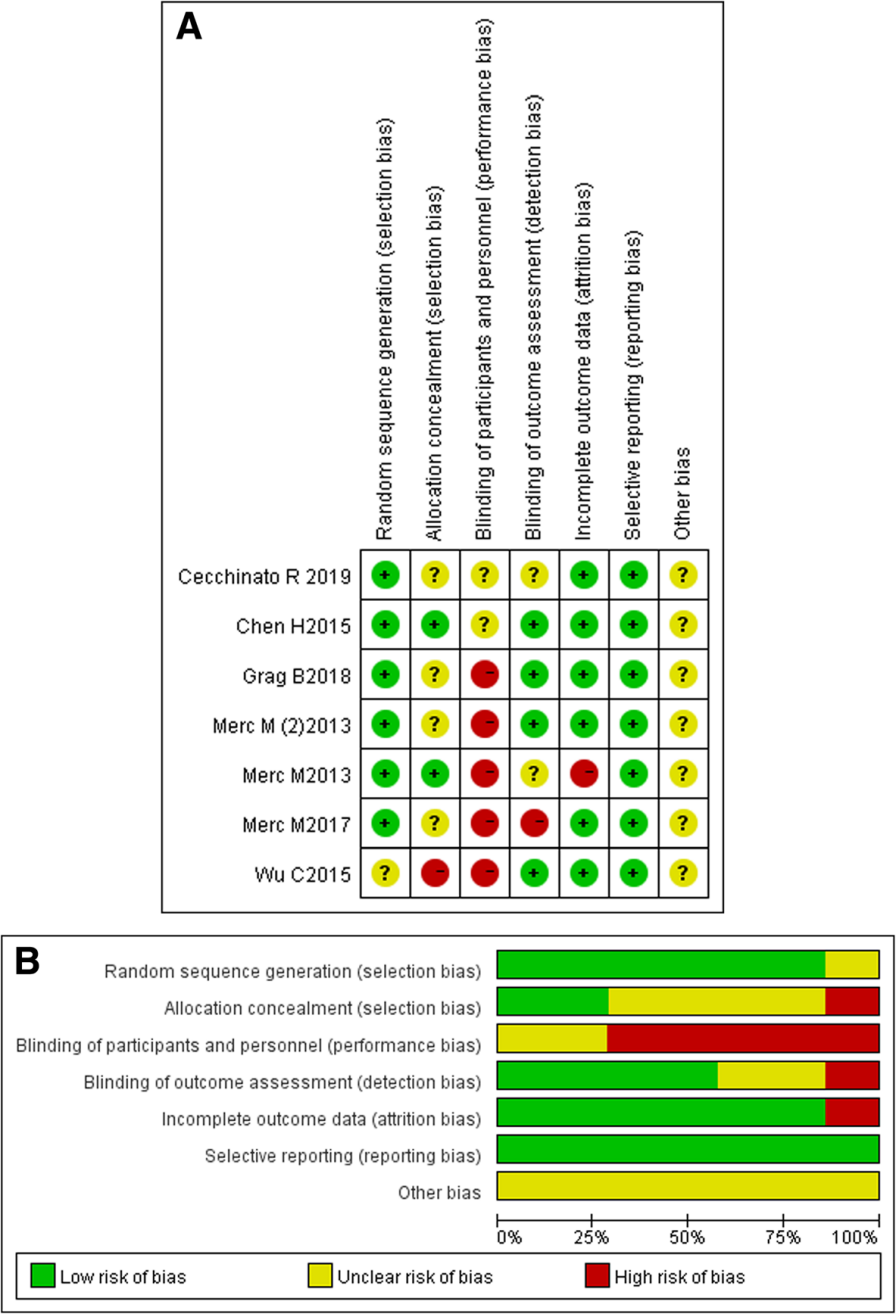
Results of the quality assessment for each included study. (**a**) Summary of the risk bias; (**b**) Graph of the risk bias

The bias of the eligible seven randomized controlled trials is summarized in Fig. 2a. Six of the seven randomized controlled trials [14, 17, 18, 22–24] had a low risk of performance bias due to the random sequence generation method, and only one study [27] had a high risk due to selection bias. Five studies [18, 22–24, 27] had a high risk of performance bias with due to the method of blinding the participants and personnel, and the other studies had an unclear risk [14, 17]. One study [24] had a high risk of detection bias, and another study [22] had a high risk of attrition bias. As illustrated in Fig. 2b, these selected studies were rated as having good quality. The quality of six nonrandomized controlled studies [19–21, 25, 26, 28] was evaluated according to the Newcastle–Ottawa Quality Assessment Scale. The results are shown in Table 2. These studies obtained scores ranging from six stars to eight stars, which indicate that the quality of the articles is good.

**Table 2:** NOS scores for six nonrandomized clinical controlled studies

Included study	Selection	Comparability	Outcome	Final score
Guo F 2017	✩✩✩	✩	✩✩	✩✩✩✩✩✩
Jiang L 2017	✩✩✩	✩✩	✩✩	✩✩✩✩✩✩✩
Liu K 2016	✩✩✩	✩	✩✩✩	✩✩✩✩✩✩✩
Pan Y 2018	✩✩✩	✩✩	✩	✩✩✩✩✩✩
Pu X 2018	✩✩✩	✩✩	✩✩✩	✩✩✩✩✩✩✩✩
Zhang Y 2015	✩✩✩	✩✩	✩✩✩	✩✩✩✩✩✩✩✩

### Meta-analysis results

#### Qualification rate of pedicle screw insertion

**Fig. 3:**
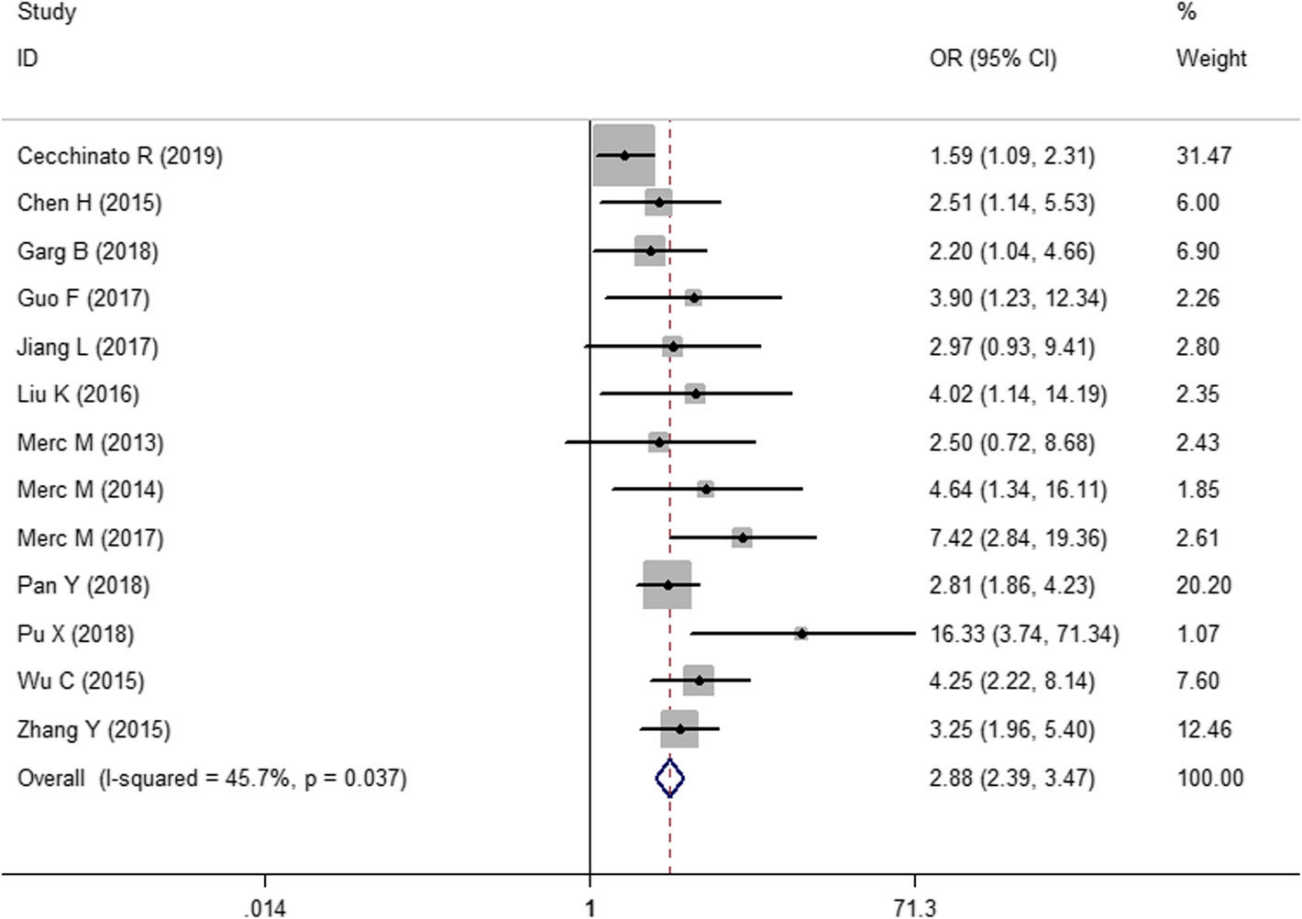
The forest figure of screw placement

As shown in Fig. 3, all studies reported that the pedicle screws were inserted by a drill guide template or the free-hand technique. These studies were not heterogeneous (*I*^2^ = 45.7%, *P* = 0.037), so the results were summarized using a fixed effects model. Considering the results of the control group, the drill guide template was more accurate than the free-hand technique in the insertion of the pedicle screws (OR = 2.88; 95% CI, 2.39~3.47).

#### Operative time

**Fig. 4:**
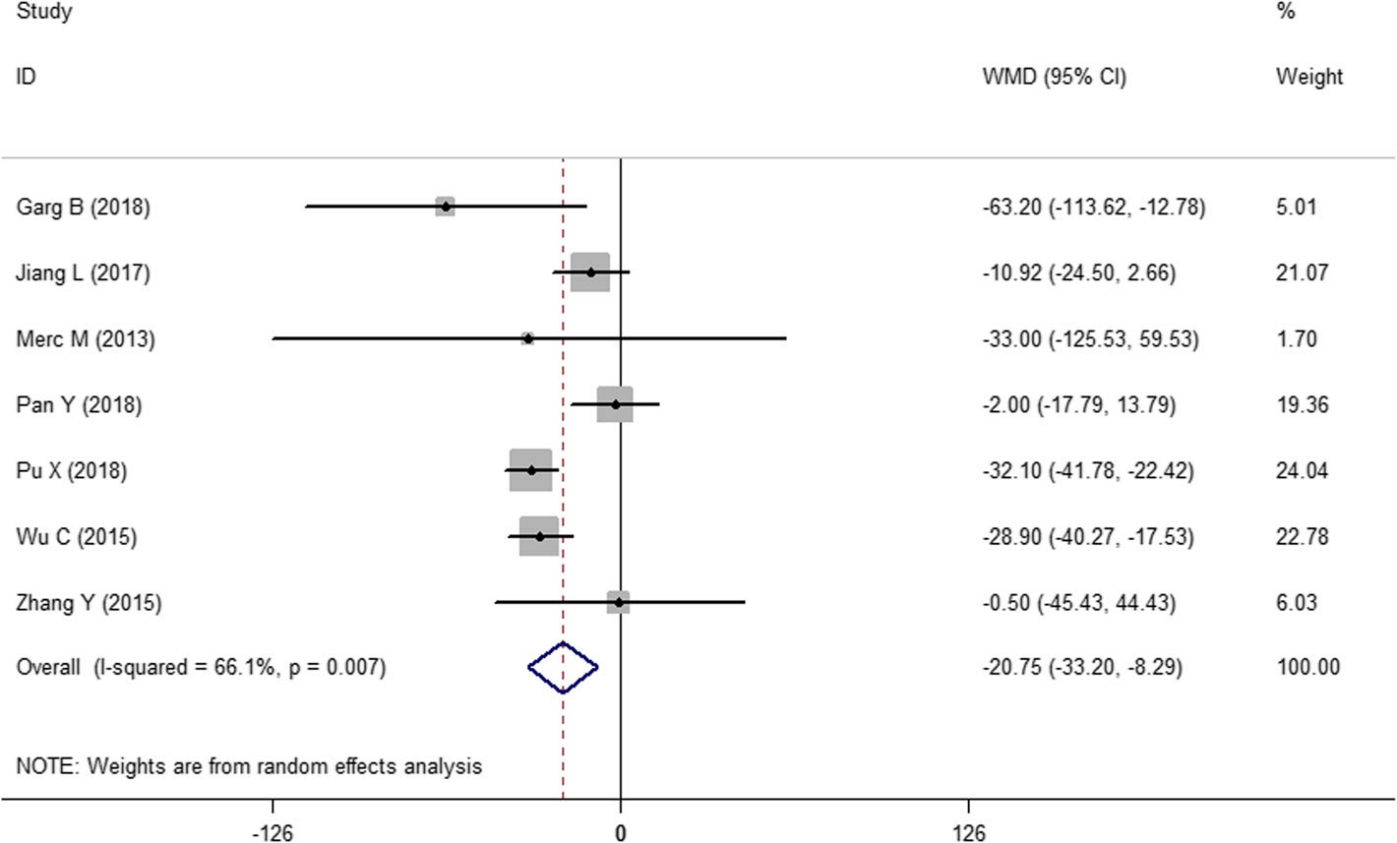
The forest figure of operative time

As shown in Fig. 4, seven of the 13 studies [18, 20, 22, 25–28] reported operative time. Since the combined results indicated significant heterogeneity among studies (*I*^2^ = 66.1%, *P* = 0.007), a random effects model was used to pool the results. The effect index WMD = − 20.75, the 95% CI (− 33.20, − 8.29), and the combined *P* = 0.001 < 0.05 indicated that there was a statistically significant difference in the operative time between the two methods. Therefore, the drill guide template reduces the operative time in pedicle screw fixation compared with that of the free-hand technique.

#### Intraoperative blood loss

**Fig. 5:**
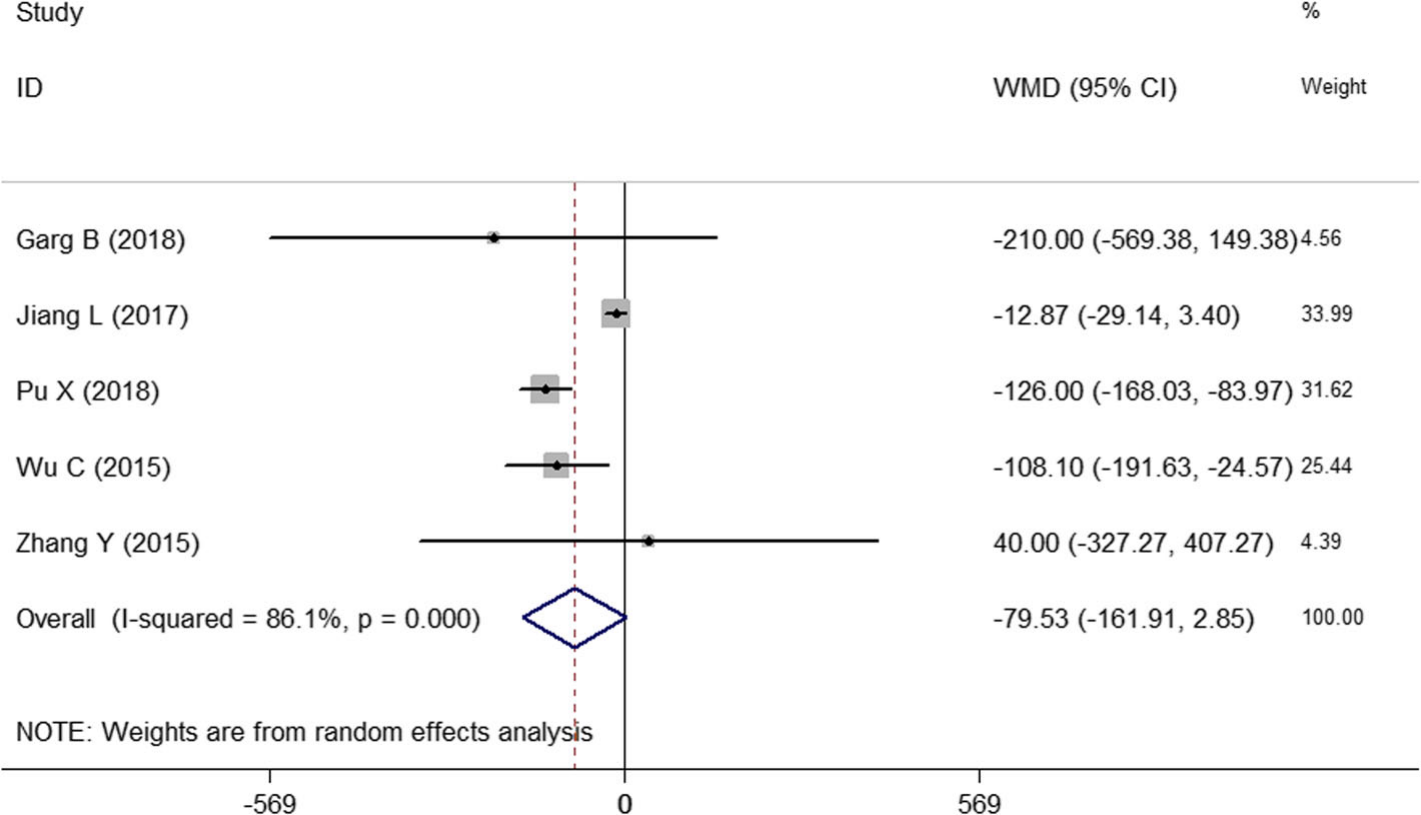
The forest figure of blood loss

As shown in Fig. 5, five of the 13 studies [18, 20, 26–28] reported intraoperative blood loss. A random effects model was used to summarize the results because the heterogeneity was significant among five studies (*I*^2^ = 86.1%, *P* = 0.000). Finally, we obtained the following results: WMD = − 79.54, 95% CI (− 161.89, 2.81), and the combined *P* = 0.058>0.05. There was no significant difference between the two groups. However, it is not clear whether the 3D-printed drill guide template resulted in less blood loss. Because the different surgical sites of patients led to different surgery outcomes, we should further analyze the sources of heterogeneity.

#### Subgroup analysis

Figure 3 shows the combined result of the number of correctly placed screws. Because *I*^2^ = 45.7%, we did not perform a subgroup analysis. However, Figs. 4 and 5 show significant heterogeneity in operative time and intraoperative blood loss because the surgical sites of the spinal segments impact the surgical effects. Moreover, the selection of the surgical position depends on the affected spinal segments. Therefore, a subgroup analysis was performed according to the different spinal segments, e.g., the first group (thoracolumbar vertebrae group) and the second group (cervical vertebrae group).

**Fig. 6:**
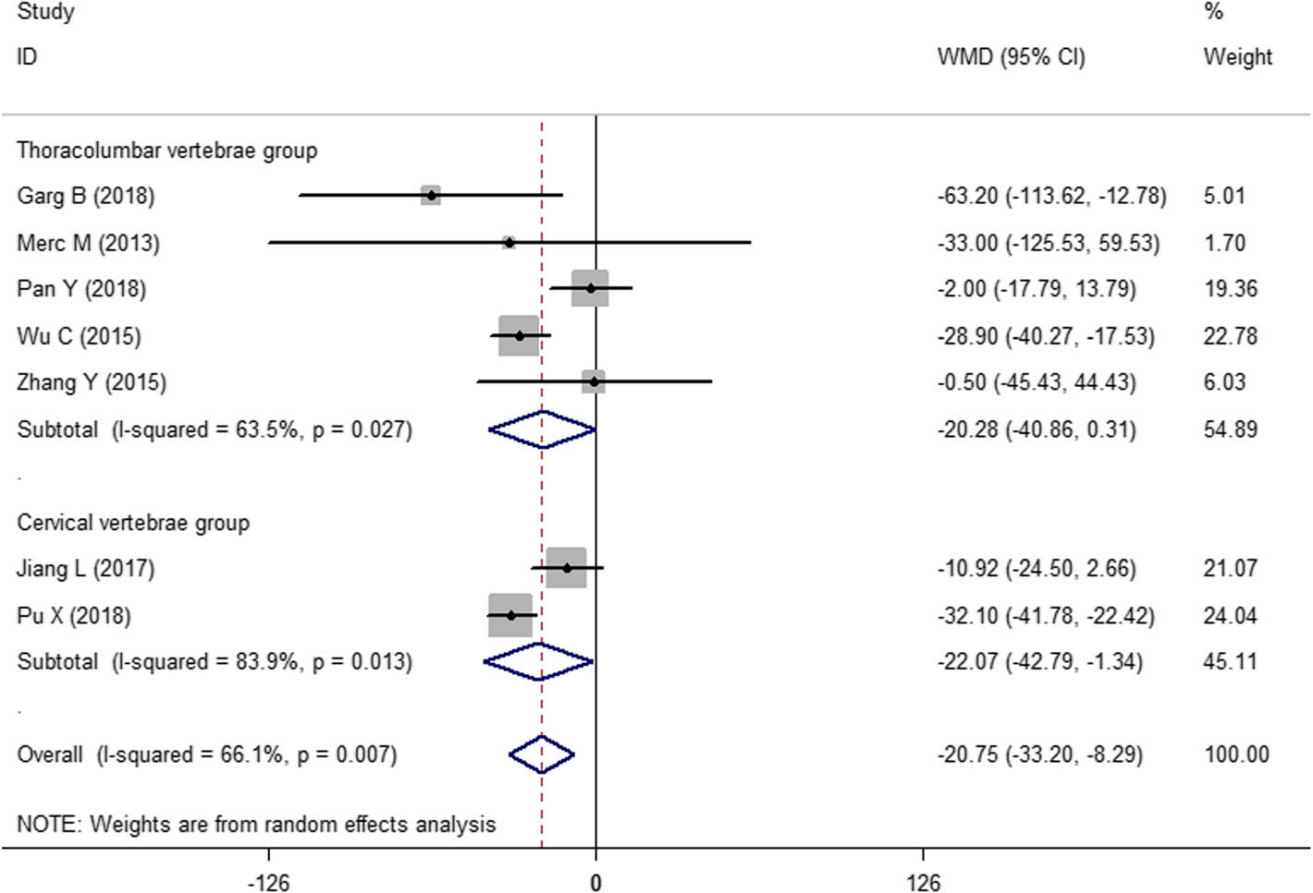
The subgroup analysis of operative time

Figure 6 shows the subgroup analysis of operative time. The two methods were statistically significant in the second group (cervical vertebrae group) (WMD = − 22.07, 95% CI, − 42.79 ~ − 1.34). Moreover, the results of the subgroup analysis indicate stability [WMD = − 20.75, 95% CI (− 33.20, − 8.29), *P* = 0.037 < 0.05].

**Fig. 7:**
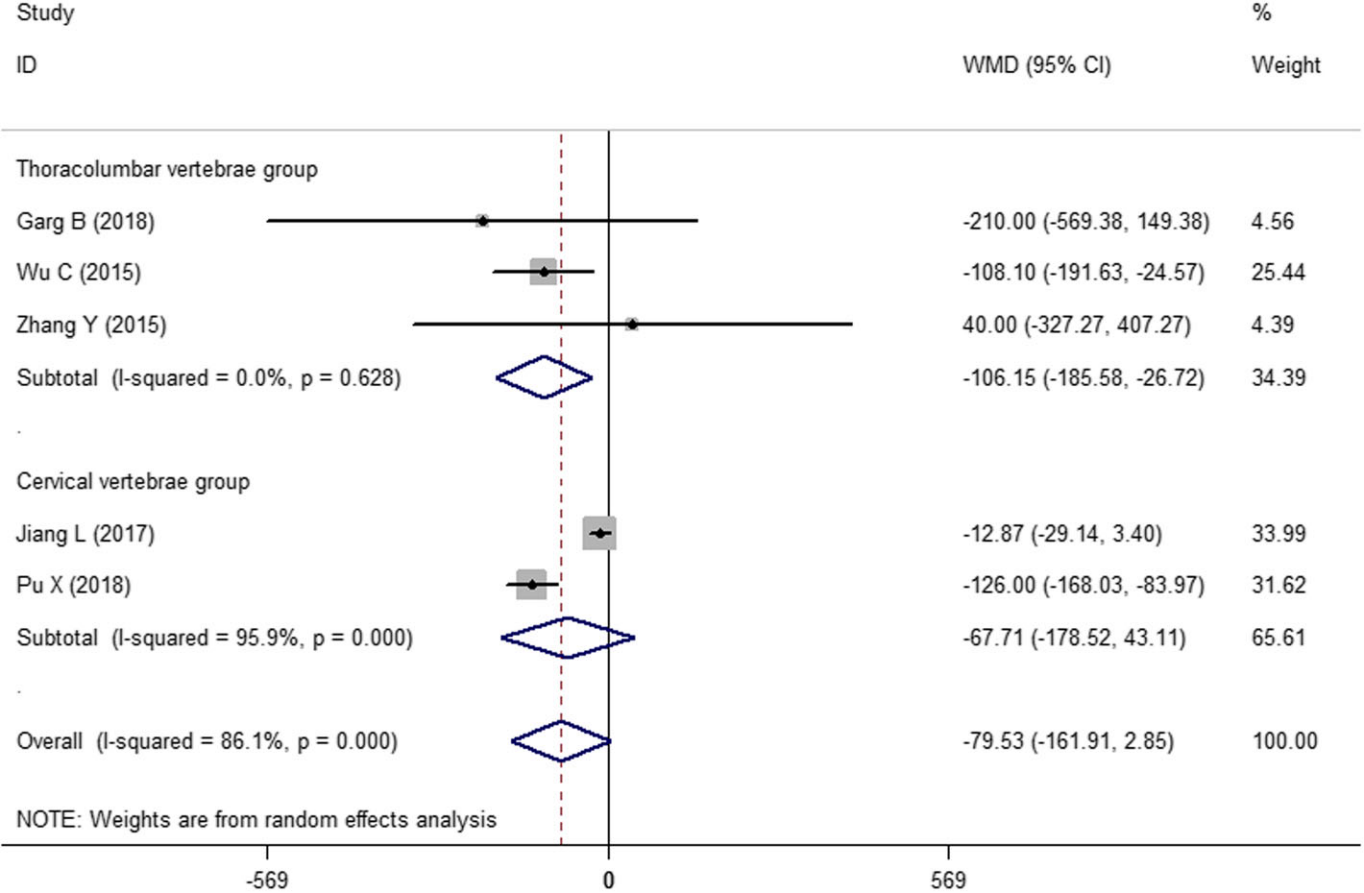
The subgroup analysis of intraoperative blood loss

As shown in Fig. 7, a subgroup analysis of intraoperative blood loss showed a significant reduction in heterogeneity in the first group (*I*^2^ = 0.00%, *P* = 0.628). The analytical result of the first group showed a statistically significant difference between the two methods [WMD = − 106.16, 95% CI (− 185.35, − 26.95), *P* = 0.009 < 0.05]. The results show that the different surgical sites or methods were sources of heterogeneity.

The drill guide template has an advantage over the free-hand technique in reducing the amount of intraoperative blood loss and the operative time.

#### Publication bias

**Fig. 8:**
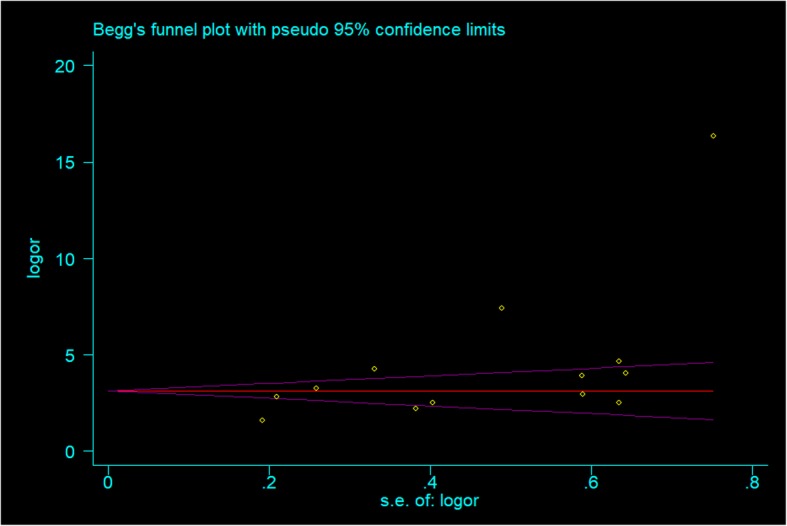
Funnel plot of publication bias

As shown in Fig. 8, the Begg funnel plot shows substantial asymmetry. Begg’s test indicated some evidence of publication bias (Begg’s test, *P* = 0.044 < 0.05; Egger’s test, *P* = 0.032 < 0.05). A reanalysis of the log-transformed data excluding the data of one study [17] showed a summary OR of 3.34 (95% CI, 2.68–4.17, *I*^2^ = 0.00%, *P* for heterogeneity = 0.469) and no evidence of publication bias among the studies (Begg, *p* = 0.086; Egger, *p* = 0.105), indicating that methodological differences in data processing may partly explain the asymmetry of the funnel plot.

## Discussion

In this meta-analysis, we evaluated the clinical efficacy (accuracy rate of the pedicle screws, operative time, and intraoperative blood loss) of two different interventions for patients with a severe spinal disease based on data from seven RCTs and six no-RCTs. This study indicates that drill guide templates significantly increase the accuracy rate of pedicle screw placement and decrease operative time and intraoperative blood loss compared with those of the free-hand technique.

The meta-analysis shows that the use of a patient-specific drill guide template that was made by 3D printing technology is superior to the traditional free-hand technique. The use of a drill guide template can reduce the perforation rate of a misplaced screw during pedicle screw fixation. This result is similar to that reported by Fan et al. Currently, pedicle screws are usually placed by the free-hand technique. However, many researchers believe that pedicle screw placement using the free-hand technique is unreliable in cases of spinal deformity [28, 29]. In addition to strengthening the surgeon’s screw placement technique, we need to use some auxiliary devices. The drill guide template using a 3D printing technique may be the best choice at present [18]. This may be the best choice because 3D printing technology and related imaging equipment can provide the spinal surgeon a three-dimensional model or imaging data of the patient’s diseased spine [30, 31]. There are different pedicle shapes, and 3D printing technology can produce many different drill guide templates that flexibly adjust the angle of the screw [7, 20, 32]. Though it improves the accuracy rate of the pedicle screw, the application of the technique is not more effective in reducing pain and disability after midterm follow-up in comparison with the free-hand technique [24].

There were significant differences between the two methods in terms of operative time in our study. The meta-analysis results showed that the drill guide template was more advantageous than the traditional free-hand technique was, and our subgroup analysis was stable and supported this result. Regarding with the intraoperative blood loss, there was no significant difference between the two groups in the total meta-analysis. However, considering that the different surgical sites and procedures can positively influence intraoperative blood loss, we thus performed a subgroup analysis. In the subgroup analysis for patients with thoracolumbar surgery, we can conclude that the drill guide template is superior to the free-hand technique in reducing intraoperative blood loss. The forest plot regarding intraoperative blood loss of cervical surgery in the subgroup analysis showed no statistical differences between the two treatments. Still, the confidence interval was skewed versus the group of free-hand technique. The confidence interval of the weighted mean difference (WMD) was wide and the possibility that the real WMD favors largely the group of drill guide template. We think that there are two factors contributing to the above results: the limited number of studies that we included and less intraoperative blood loss in the cervical surgery [33, 34]. This study illustrated the advantages of the drill guide template, which can accurately and effectively increase the accuracy rate of pedicle screw placement and decrease the risk of postoperative complications, such as shorten the operative time and reduce intraoperative blood loss. However, the drill guide template also has some disadvantages. First, to insert the pedicle screw in the ideal position during a fixation surgery, the spinal surgeon must clean or cut the soft tissue or bone tissue near the implantation site. This process will increase the chance of bleeding during surgery or relatively prolong the operative time [35]. Second, in minimally invasive spinal surgeries, there are problems such as a crowded operative space and unclear vision caused by surrounding tissue structures, which inevitably increases the surgeon’s requirements for placing the screw [36]. Other issues associated with applying the drill guide template include template disinfection, template deformation, and the need to take time to learn the 3D printing software [35, 37].

The advantage of this meta-analysis is that the included studies do not have animal experiments or cadaver specimen experiments. Although previous studies have compared the accuracy rate of pedicle screw placement, their inability to simulate the true state of the clinical trials is their greatest drawback. Second, the secondary outcome of our meta-analysis determined whether the 3D printed drill guide template shortens operative time and reduces intraoperative blood loss. This outcome provides a reference for the selection of surgical methods in the future. For example, when a patient has a disease that cannot tolerate a long period of surgery, the spinal surgeon may have to consider other methods or formulate a contingency plan prior to the surgery.

However, there are also some limitations of our study. First, the number of articles and the sample size were small, which may affect the overall results. Second, we did not separate subjects by age, sex or disease type. Other factors affecting heterogeneity were not analyzed, such as we only scarcely mention the heterogeneity of the study population. Moreover, because the patients included these studies had different diseases, the results may be affected and need to be interpreted with caution. Finally, the number of eligible studies was obviously insufficient, as it included only seven randomized controlled trials; thus, the evidence provided by the meta-analysis may be inadequate. Moreover, there was publication bias in this meta-analysis, so we should interpret these results with caution. We hope that future work will include a larger number of studies and randomized clinical controlled trials with longer follow-up times and groups of patients with the same disease; a multicenter, prospective investigation should be considered. Meanwhile, there is a need for a scientific comparison of this technique with more methods, like computer navigation and robot arms.

## Conclusion

In conclusion, the drill guide template significantly increases the accuracy rate of the pedicle screw and decreases operative time and interoperative blood loss compared with those of the free-hand technique. However, because our study has significant publication bias and other limitations, stricter studies with a larger sample size will help analyze these issues in the future.

## Data Availability

The datasets generated and analyzed during the current study are available from the corresponding author on reasonable request.

## Abbreviations

CIs: Confidence intervals
CT: Computed tomography
JOA: Japanese Orthopaedic Association Score
NOS: Newcastle-Ottawa Scale
ODI: The Oswestry Disability Index
ORs: Odds ratios
RCTs: Randomized controlled trials
SD: Standard deviation
TN: Total number
UC: Unclear
VAS: Visual Analogue Scale/Score
WMDs: Weighted mean differences
